# Supplementary figures and images for: Six-Lead Electrocardiography Enables Identification of Rhythm and Conduction Anomalies of Patients in the Telemedicine-Based, Hospital-at-Home Setting: A Prospective Validation Study
Source: Sensors (Basel). 2023 Oct 14;23(20):8464. doi: 10.3390/s23208464 (PMC10611340; doi:10.3390/s23208464)

Supplementary Figure 1. Illustration of use of the 6-lead KardiaMobile device.

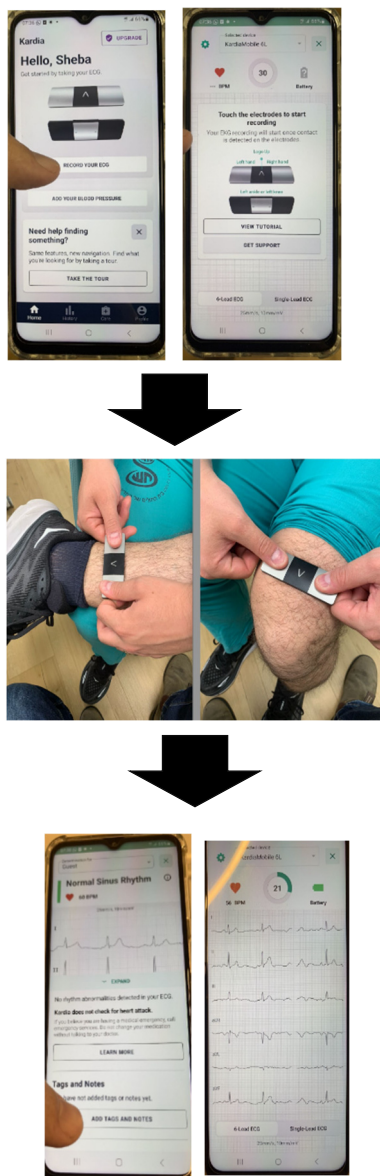

Supplement: Supplementary file 1 [file sensors-23-08464-s001.zip › sensors-2598235-supplementary.pdf]
